# Toward a digital sociology of artrepreneurship: understanding artistic entrepreneurship in the platform society

**DOI:** 10.3389/fsoc.2026.1961364

**Published:** 2026-10-09

**Authors:** Maria Susan Mathew, Ajimon George, Emilda K. Joseph, Anoop Tom Thomas, Ajo Andrews, Soumya Varghese

**Affiliations:** 1 Marian College Kuttikkanam Autonomous, Kuttikkanam, Kerala, India; 2 St. Dominic’s College, Kanjirapally, Kerala, India; 3 Catholicate College, Pathanamthitta, Kerala, India; 4 Bharata Mata College (Autonomous), Thrikkakara, Kerala, India

**Keywords:** artistic entrepreneurship, artrepreneurship, cultural production, digital labor, digital sociology, platform governance, platform society, social media

## Abstract

Artistic entrepreneurship is a rapidly changing field due to the growing dominance of social media. Digital technologies have opened up new avenues for artists to produce, market, and monetize their creative work, and have also remediated the social mechanisms, identities, and structures through which artistic value is created and recognised. Research on social media has so far focused on entrepreneurial, marketing and technological aspects, particularly platform adoption, digital marketing and economic performance. As a result, little attention has been given to understanding artrepreneurship as a socially constructed activity, influenced by platform infrastructures, algorithmic governance, digital labour, identity performance and networked social relationships. This Perspective calls for a reorientation from viewing social media as a communication medium toward understanding platforms as digitally mediated institutional environments that shape artistic production, visibility, legitimacy, and entrepreneurial opportunity. This article proposes a conceptual framework that integrates insights from digital sociology, platform studies, social capital theory, and Goffman’s dramaturgical perspective to understand contemporary artistic entrepreneurship: Digital Sociology of Artrepreneurship (DSA). DSA conceptualises artrepreneurship as a dynamic process in which individual agency interacts with platform-mediated social structures, with artistic and entrepreneurial outcomes shaped by visibility, identity performance, networked relations and strategic engagements within digitally mediated ecosystems. A research agenda for the future and theoretical implications for sociology, cultural studies, entrepreneurship, and research on a digital society are presented in the conclusion.

## 1 Introduction

The platformization of society has substantially changed the context in which artistic entrepreneurship takes place. While galleries, publishers, exhibitions and other cultural intermediaries traditionally act as the bridges between artists and audiences, digital platforms like Instagram, TikTok, YouTube, Etsy, and Behance have emerged as key infrastructures that artists use to produce, circulate, and commercialize creative work. As a result, barriers to entry have been reduced, creating opportunities for artists to build global audiences, develop personal brands, diversify income streams, and transform creative practices into sustainable entrepreneurial careers (Abidin, 2020; Duffy, 2017). This has created the need to embed artistic entrepreneurship into digitally mediated spaces where creative production, social interaction and economic exchange all take place.

The term “artrepreneurship” was coined to refer to a combination of artistry and entrepreneurial skills needed to create viable careers in the creative industries (Chang and Wyszomirski, 2015; Essig, 2017). In this article, the terms are used with specific distinctions. The term “artistic entrepreneurship” is used in an inclusive way and it is a field of research that has emerged from the field of arts entrepreneurship known as “entrepreneurial activity in and around artistic practice” (Chang and Wyszomirski, 2015; Essig, 2017). In this context, “artrepreneurship” is a more precise term that describes the entrepreneurial process in which artists engage in artistic production and entrepreneurial endeavours in today’s digital and platform-mediated contexts. An entrepreneur who engages in such entrepreneurial activity is called an “artrepreneur.” Thus, artistic entrepreneurship is used as the broader field, artrepreneurship refers to the entrepreneurial process examined in this context, and artrepreneur refers to the artist engaged in that process. Early scholarship focused on entrepreneurial skills, creativity, opportunity identification, market skills and entrepreneurship development (Bridgstock, 2013; Pollard and Wilson, 2014). With the advent of digital platforms as the focus of creative practice, more research looked at how social media can help artists promote their work, connect with audiences, build professional networks and monetise their artistic output outside of institutional structures (Duffy, 2017). To date, however, most of the scholarship examining digital platforms has focused on how they can be used for entrepreneurial work rather than how they create space for entrepreneurial work. This research, however, has tended to take a more technological or marketing lens, seeing digital platforms less as a space to shape entrepreneurial practice and more as a tool to make use of it.

There is now some emerging empirical evidence that a more complex reality exists. As part of this study, a phenomenological exploration of artrepreneurs revealed that social media is not simply a medium for promotion but a multidimensional space in which artistic expression, audience interaction, networking, identity construction, perceived opportunities, and entrepreneurial outcomes are negotiated throughout interactions on social media platforms (George and Mathew, 2025). In recent years, conceptual research on artificial intelligence in artistic entrepreneurship also posited that AI has an additional impact on platform-based practices by changing how artists create, how they are considered authentic, how they are visible in the world, and how they make entrepreneurial decisions, and it has raised new ethical, institutional, and governance issues about authorship and creativity (George and Susan Mathew, 2025). These studies imply that, rather than being only about entrepreneurial abilities, contemporary artistic entrepreneurship is growingly defined by engagements with digital infrastructures.

Even with these developments, research on social media and artificial intelligence mostly focuses on entrepreneurship, marketing or communication or technology. These methods have produced important findings on the implications of digital branding, audience interaction, entrepreneurial behaviour, and technology adoption, but have tended to lessen the focus on larger sociological processes shaping, organizing and governing artistic visibility, identity, legitimacy and opportunity through platform infrastructures, algorithmic governance, institutional rules, and networked interactions. Digital platforms are not just the enablers of entrepreneurship, they are becoming the institutional contexts of entrepreneurship.

The need is addressed by digital sociology that offers an important theoretical base. Digital sociology sees platforms as institutions that shape and mediate interaction, structure power and participation, and reshape everyday life, instead of being neutral tools (Couldry and Hepp, 2016). In the same way, platform studies show how algorithmic logic and data infrastructures and governance frameworks are increasingly shaping cultural production and the allocation of visibility and recognition (Poell et al., 2021; van Dijck et al., 2018). In this view, artistic entrepreneurship is not an economic phenomenon per se, but it is a social phenomenon that takes shape over time via interaction between different populations, including artists and audiences, algorithms and platform institutions.

Following this sociological approach, some recent studies have started conceptualising the Digital Strategic Engagement as a communicative practice consisting of various dimensions, including strategic platforms, artistic expression, strategic audiences, strategic networking and strategic interaction within platform ecosystems (Mathew et al., 2026). This work contributes to the understanding of how artists strategically interact with digital platforms, but the definition of Digital Strategic Engagement is mostly one based on measuring behaviour. It is still needed to have a broader theoretical account of how such practices manifest themselves in digitally mediated social structures.

In view of this, this Perspective proposes the Digital Sociology of Artrepreneurship (DSA) as a conceptual framework for understanding artistic entrepreneurship in the platform society. DSA conceptualises artistic entrepreneurship as a socially mediated process influenced by institutional structures, algorithmic governance, identity performance, networked social relations, and individual agency, rather than treating social media and digital technologies merely as entrepreneurial tools. The synthesis of digital sociology, platform studies, embeddedness, Bourdieu’s theory of capital and Goffman’s dramaturgical perspective, is used to illuminate how artistic visibility, legitimacy and entrepreneurial opportunities arise as a result of the interplay between artists and digitally mediated social structures. The rest of the article first outlines the shortcomings of the previous approaches and then elaborates on the five dimensions of the DSA framework, and lastly reflects on their implications for sociological research on entrepreneurial artistic practice.

## 2 Why existing perspectives on artrepreneurship are no longer sufficient

The term artrepreneurship has been introduced to describe the combination of artistic entrepreneurial skills and the aim of sustainable careers in the creative industries (Chang and Wyszomirski, 2015; Essig, 2017). In the early days, entrepreneurial skills, opportunity recognition, innovation, marketing and venture creation were the main focuses of entrepreneurship scholarship (Bridgstock, 2013). As the social media landscape grew, research was even more revealing of the ability of digital platforms to extend audiences, cultivate personal brands, and monetise creative content beyond the cultural sector (Duffy, 2017).

Although these are important contributions to the field, the current literature has mainly approached social media from the perspective of the entrepreneur or marketing. This view neglects the social role of platforms as social infrastructure that governs social interaction, regulates visibility, frames participation, and thus affects cultural legitimacy. Digital platforms are not merely means of enabling entrepreneurship; they are also the environments in which entrepreneurship takes place.

Entrepreneurship is related to networks, institutions and power relations from a sociological point of view (Granovetter, 2008). Platform society and algorithmic systems, platform governance, and data infrastructures influence access to audiences, opportunities, and economic opportunities (Poell et al., 2021; van Dijck et al., 2018). Therefore, to succeed in art, creativity and entrepreneurship skills are not only essential, but, the knowledge of how to maneuver digitally mediated social landscapes is essential.

Authenticity, engagement, understanding of audience data, keeping up with changes in platforms, and entrepreneurial identity are essential traits for today’s artists. Artistic production is thus a process of self-presentation, interaction and acts of community engagement in the digital realm.

However, the existing theories of the transformation only account for part of this change. Algorithmic intermediation of social structures is not sufficiently discussed in the study of entrepreneurship, marketing research is focused primarily on the involvement of consumers, and the study of creative industries does not seriously consider the digital infrastructures as institutions of culture governance. These limitations suggest the need for a sociological framework that can account for the influence of platform-mediated interactions on artistic entrepreneurship.

Artrepreneurship is proposed in this article as a socially embedded practice that is not solely economic, but rather is an ongoing process of negotiating identities, visibility, social relationships, and entrepreneurial opportunities between artists, audiences, algorithms and platform infrastructures.

## 3 Toward a digital sociology of artrepreneurship

The above discussion highlights that the concept of entrepreneurship in the context of contemporary art cannot be explained and understood by using only a traditional theory of entrepreneurship and a digital marketing theory. These viewpoints, although useful for explaining how artists identify opportunities and use social media to market their artistic work, do not offer much understanding about the ways that digital technologies alter the social contexts in which artistic entrepreneurship is practiced. This article therefore proposes the Digital Sociology of Artrepreneurship (DSA) as a conceptual framework for placing artistic entrepreneurship within the broader socio-economic context of the platform society.

Digital Sociology of Artrepreneurship (DSA) is specifically understood as a conceptual framework for analysing artistic entrepreneurship as a socially mediated platform practice. DSA is not intended as a new field of research, or a stand-alone sociological theory, but rather a tool for integrating insights from digital sociology, platform studies, social embeddedness, social capital and dramaturgical theories to explain how artistic entrepreneurship is constituted through the interplay of platform structures, algorithmic processes, social relations, identity performances and entrepreneurial agencies. Accordingly, this article uses DSA as a conceptual framework for analysing artrepreneurship as a platform-mediated social process in which structural conditions, algorithmic mechanisms, relational processes, identity construction, and individual agency are mutually constituted.

Digital Sociology of Artrepreneurship is a conceptual framework that aims to examine the interplay of digital platforms, algorithmic systems, networked communities, and platform governance in the process of creating artistic identity, artrepreneurial practices, and creative livelihoods. Instead of seeing these factors as separate influences, DSA considers these factors to be interdependent and examines how they collectively shape the opportunities, restrictions, relationships and forms of agency within which artistic entrepreneurship is manifested. DSA therefore conceptualises social media platforms not as external technological resources that artists simply use, but as constitutive social environments in which artistic production, interaction, visibility, identity, and entrepreneurial exchange are organised. Artistic entrepreneurship is a concrete process of the continuous interplay between individual agency and digitally mediated social structures.

This perspective is consistent with the view that digital technologies are not merely tools that facilitate social interaction but are constitutive of the social environments in which social life is organized and experienced (Orlikowski, 2007). In a platform society, artists engage in algorithmic ecosystems that are shaped by the norms of platforms, recommendation systems and visibility and engagement metrics (van Dijck et al., 2018). These mechanisms not only drive the spread of artistic content but also reputation, audience trust and the legitimacy of the entrepreneur.

The five dimensions operate at different analytical levels and functions, but they are conceptually integrated within a single structure–mechanism–agency process. Platform Embeddedness describes the structural context of artistic entrepreneurship, Algorithmic Visibility is a platform-mediated mechanism of selective distribution of attention, recognition and access, Performative Artistic Identity is a process of identity construction and negotiation within the context of a platform, Networked Social Capital is the relational resources and recognition created by interactions with audiences, peers, collaborators and communities, and Digital Strategic Engagement refers to the process through which artists interpret, respond to, adapt to, and negotiate these structural, algorithmic, identity and relational conditions. They should therefore not be understood as five parallel causal variables, but as five interconnected analytical components through which the platform-mediated artistic entrepreneurship can be understood.

Conceptually, the relationship can be understood as recursive rather than strictly linear. Algorithmic visibility is conditioned by platform embeddedness, which provides the institutional and infrastructural conditions of participation. Artists respond to these conditions through identity performance, the cultivation of networked social capital and digital strategic engagement. These responses can affect subsequent visibility, identity construction and network relations. The five dimensions are thus a dynamic structure–agency configuration in which platforms condition action, artists responds strategically, and their responses are constantly shaped by the algorithmic and relational feedbacks.

Artificial intelligence can be understood within DSA as an intensifying layer of platform mediation rather than as a separate dimension. AI-enabled recommendation, content-generation, moderation and analytics systems can influence algorithmic visibility, while generative AI can transform artistic identity, creative labour and strategic engagement, highlighting further sociological questions of authorship, authenticity, inequality, skill and control over creative production (George and Mathew, 2025). AI can also reshape platform embeddedness by altering governance, moderation and infrastructural arrangements, while recommendation and generative systems may influence the formation and maintenance of networked social capital by changing patterns of interaction, connection and community formation.

The scope of DSA is therefore bounded to artistic entrepreneurship in digitally mediated and platform-dependent environments. It is not proposed as a universal explanation of all forms of artistic entrepreneurship. Its analytical value is greatest where platform infrastructures, algorithmic mediation, networked interaction and digitally performed identities are central to the organisation of artistic and entrepreneurial activity.

Digital Sociology of Artrepreneurship does not merely combine existing sociological theories but synthesizes them into a unified framework for understanding artistic entrepreneurship in the platform society. Digital sociology explains how digital technologies restructure social life, while platform studies reveal how platforms function as institutional infrastructures that govern participation and visibility. Granovetter’s concept of embeddedness situates entrepreneurial activity within socially structured networks, Bourdieu’s theory of capital explains how recognition and legitimacy are accumulated through digitally mediated relationships, and Goffman’s dramaturgical perspective illuminates the performative construction of artistic identities online. Together, these perspectives demonstrate that artistic entrepreneurship emerges through the continuous interaction between individual agency and platform-mediated social structures. The five dimensions of DSA operationalize this sociological perspective.

DSA also connects artistic entrepreneurship with the sociology of digital labour. Platformization is not only a transformation of how artistic work accesses markets, but also of how creative work is organised, assessed and maintained. Artists increasingly perform activities beyond creative production, including content creation, self-presentation, audience management, networking, analytics interpretation, and continuous adaptation to the platform changes (Duffy, 2017; Poell et al., 2021).

This also raises questions central to the sociology of work, including the distribution of control and autonomy, the intensification of creative labour, occupational insecurity, and the expansion of unpaid or aspirational labour required to maintain platform visibility. Platform governance can therefore operate as a form of indirect organisational control, as artists adapt their work practices to changing algorithmic expectations while remaining formally independent. DSA consequently connects entrepreneurial agency with the organisational and labour conditions under which digitally mediated creative work is performed.

The theoretical novelty of DSA lies not in introducing entirely new sociological concepts, but in specifying how established mechanisms operate together within platform-mediated artistic entrepreneurship. Creative entrepreneurship explains opportunity recognition, resource mobilisation and value creation; platform studies explain infrastructure, governance and algorithmic mediation; digital-labour research highlights the labour required to remain visible and competitive; and cultural-production perspectives explain the organisation of cultural production. DSA connects these insights within a single sociological account of how platform structures condition visibility and opportunity, how artists construct and negotiate identities, how networked relationships generate social capital, and how artists exercise strategic agency within these conditions. Its distinctive contribution is therefore relational: DSA explains artrepreneurial outcomes as emerging from the recursive interaction of platform structures, algorithmic visibility, identity performance, networked social capital and strategic agency rather than treating these as separate explanations (see Figure 1).

**Figure 1:**
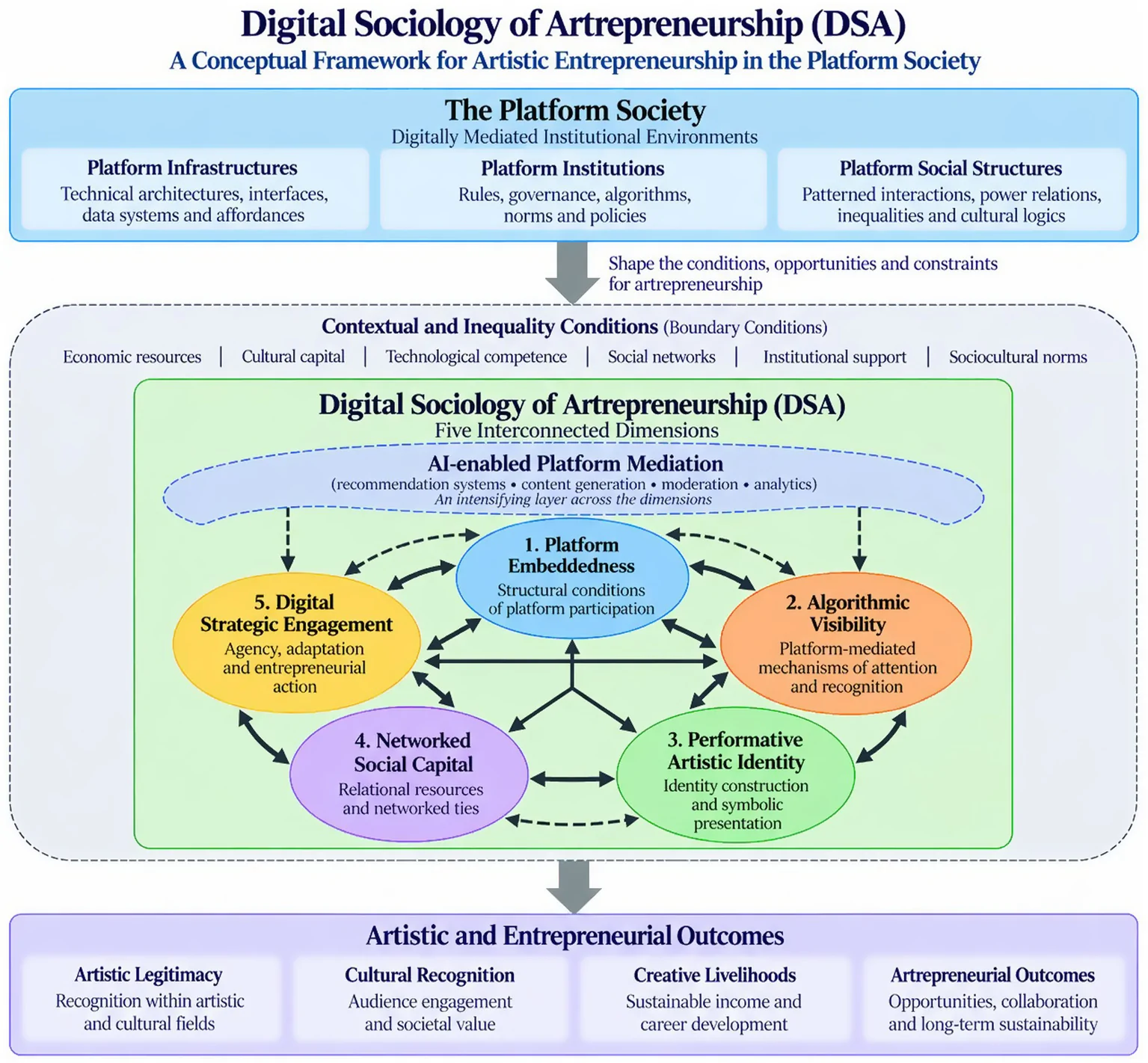
Conceptual framework of the digital sociology of artrepreneurship (DSA).

DSA conceptualises artistic entrepreneurship as a platform-mediated social process involving structural conditions, platform-mediated mechanisms, identity construction, relational resources and individual agency. The five dimensions— Platform Embeddedness, Algorithmic Visibility, Performative Artistic Identity, Networked Social Capital, and Digital Strategic Engagement—are conceptually distinct but mutually recursive rather than independent causal predictors. AI-enabled platform mediation operates across these dimensions, while contextual and inequality conditions function as boundary conditions. Together, the five dimensions constitute the structure–mechanism–agency configuration through which artistic legitimacy, cultural recognition, creative livelihoods and artrepreneurial outcomes are negotiated.

### 3.1 Platform embeddedness

Economic action is not autonomous, but is very much part of social structures (Granovetter, 2008). Artistic entrepreneurship in the platform society is increasingly embedded in digital platforms that govern how audiences access content, how artistic work becomes visible in the cultural field, and how it can generate economic opportunities. Digital platforms are not just communication technologies, but institutional ‘environments’ that structure participation, norms of interaction and distribution of recognition.

It is significant to differentiate infrastructure, institution and social structure. Platforms are infrastructures that offer the technical architectures, interfaces, data systems and affordances through which content is created, circulated, prioritized, and consumed. They are also institutions with rules, governance mechanisms and norms that govern participation and establish the conditions under which cultural activity takes place. Through repeated interactions within these infrastructures and institutional arrangements, patterned social relationships, advantages and constraints can emerge among artists, audiences and collaborators. Thus, infrastructure provides certain forms of action, institutional rules structure these acts, and repeated interactions shape opportunities and inequalities (Poell et al., 2021; van Dijck et al., 2018). These conditions are also shaped by differences in resources and power. Artists with unequal access to digital skills, financial resources, cultural capital, institutional support, and established networks may experience the same platform environment differently. Platform embeddedness therefore incorporates not only the conditions of participation but also the unequal capacities through which artists can navigate and benefit from those conditions.

Platform infrastructures increasingly evaluate and distribute artistic content through algorithmically mediated systems of ranking, recommendation and engagement, which are different from the traditional cultural intermediaries like galleries or publishers. Artistic practice is then situated in an institutional framing that is co-produced in the process of the creative work’s visibility, legitimacy and opportunities, within interactions between the creator, the audience, and platform governance. Platform embeddedness therefore refers to the extent to which creative work is organised within socially mediated environments whose infrastructures, institutional rules and governance arrangements shape participation and opportunity.

### 3.2 Algorithmic visibility

In the platform society, algorithmic systems have become increasingly influential in the organisation of artistic visibility, although visibility and recognition have long been shaped by cultural intermediaries and social processes. Recommendation algorithms can function as contemporary cultural intermediaries, focusing attention according to platform-specific signals like interaction, consistency and engagement (Poell et al., 2021). Rather than distributing visibility neutrally, these systems selectively distribute attention, circulating and potentially amplifying the work of some artists and their practices more than others in digital cultural spaces. Algorithmic visibility can therefore be seen as a possible avenue of stratification through the platforms. This does not mean that algorithmic visibility directly determines artistic success or that every difference in visibility constitutes social stratification. Rather, stratification may emerge when repeated and uneven patterns of attention and recognition systematically differentiate artists and artistic practices across platform-specific ranking, recommendation, moderation and engagement systems. These disparities in visibility can shape access to audiences, collaboration opportunities, symbolic recognition and ability to transform visibility into economic and/or career opportunities. From a sociological perspective, algorithmic visibility becomes meaningful if repeated and patterned differences in exposure lead to lasting differences in recognition, legitimacy, participation, and/or opportunity.

It can be represented as a sequence of visibility–recognition–opportunity. Algorithmic selection has an initial impact on the likelihood that specific content of the artistic product will be seen by viewers. Differential exposure may then affect the attention and interaction of the audience and their sense of popularity, leading to recognition and legitimacy within networked communities. When this recognition is translated into followers, collaborations, commissions, sales, memberships or other opportunities, visibility can contribute to entrepreneurial outcomes. The process is therefore cumulative but relational. Algorithmic visibility does not itself create artistic value; rather, it can alter the conditions under which artistic value becomes socially visible and potentially actionable in economic or professional terms.

This formulation also recognises that algorithmic visibility interacts with pre-existing cultural, social, and economic inequalities. Artists enter platform environments with unequal access to skills, networks, resources, reputational capital, and institutional support. Platform-mediated visibility may therefore reproduce, intensify, or occasionally disrupt existing inequalities rather than constituting an entirely independent hierarchy. DSA consequently treats algorithmic visibility as one mechanism through which stratification may be reproduced within platformized artistic entrepreneurship.

### 3.3 Performative artistic identity

Digital platforms have created conditions in which artistic self-presentation becomes continuous and publicly observable. In digitally networked environments, artists continuously negotiate multiple roles as creators, entrepreneurs, educators, influencers, and community participants. Artistic identity is no longer expressed solely through creative production but is socially constructed through ongoing interactions with audiences, platform norms, and algorithmic expectations (Forsyth, 2024). This can create tensions between artistic originality and the need for platform visibility, as creators negotiate between maintaining creative authenticity and adapting their practices to platform algorithms. Artistic identity, therefore, needs to be conceived as a socially negotiated and institutionally mediated performance and not as an individual attribute. Performative Artistic Identity and Digital Strategic Engagement are analytically distinct but mutually related dimensions. Performative Artistic Identity focuses on what the artists build and shape and how they negotiate their identity in relation to the audience and the demands of the platform, which involves presentation and negotiation of the artistic self. Digital Strategic Engagement has to do with “what artists do” when it comes to their platform context: that is, the purposive selection, adaptation, communication, interaction and negotiation practices in which they seek to achieve artistic and entrepreneurial goals. The former concerns identity construction and symbolic presentation, whereas the latter concerns strategic agency and action. For instance, an artist may alter the presentation of their artistic identity to maintain authenticity or credibility while also adapting how they post, interact with their audience, network, or select platforms in order to achieve greater visibility or opportunity.

### 3.4 Networked social capital

The idea of social capital (Bourdieu, 2008) offers a useful way of thinking about the ways in which social networks create opportunities for access to resources, legitimacy, and opportunities. In platform ecosystems, social capital is increasingly formed and maintained through digitally mediated interactions among creators, audiences, collaborators, and communities. Networked social capital is not an indicator of the size of an audience, but rather is the product of trust, reciprocity, shared recognition and ongoing interaction that increases an artist’s social standing in platform communities. From this point of view, cultural legitimacy is therefore understood as a process co-produced through networks of social relations rather than as the result of individual success alone.

### 3.5 Digital strategic engagement

Digital Strategic Engagement is the process whereby individual agency is realized within platform-mediated social structures. This dimension acknowledges artists as more than merely subjects of algorithmic systems; it recognises them as active participants who interact with platform’s norms, adjust to algorithmic shifts, establish communities, and negotiate opportunities and constraints in the pursuit of creative work. Agency hence becomes relational, emerging through the ongoing interactions among creators, audiences, algorithms and institutional arrangements.

Recent studies have conceptualized Digital Strategic Engagement in Artrepreneurship as a multidimensional communicative practice involving strategic platform use, artistic expression, audience engagement, networking, and adaptive interaction within platform environments (Mathew et al., 2026). Digital Strategic Engagement does not simply refer to the uses of digital platforms, but rather to the ways in which creators strategically utilise platform affordances to maintain their artistic identities, develop social relationships and respond to opportunities and limitations in digitally mediated environments (Mathew et al., 2026).

From this perspective, Digital Strategic Engagement is conceptualised not only as a communicative capability but also as an agentic dimension of platform-mediated artistic entrepreneurship within the Digital Sociology of Artrepreneurship framework. Performative Artistic Identity concerns how artists construct and negotiate their identities within platform environments, whereas Digital Strategic Engagement concerns how they strategically engage with these environments to pursue artistic, relational and entrepreneurial goals. The four dimensions outlined above define the institutional, algorithmic, identity and relational context of artistic entrepreneurship, while Digital Strategic Engagement refers to how artists engage with and respond to these dimensions in their everyday digital practices. This distinction places agency within a relational space rather than an autonomous one: agency is exercised but is also enabled, limited, and constantly transformed by platform infrastructures and algorithms, identity expectations and expectations, and networked relationships.

## 4 Implications for sociology and a future research agenda

The sociological contribution of DSA lies in repositioning the artrepreneur within digitally mediated institutional environments. Rather than treating social media as a neutral communication or marketing tool, DSA conceptualises platforms as social structures that shape identity, visibility, relationships and entrepreneurial opportunity. This connects the sociology of digital platforms with the sociology of cultural production, work and entrepreneurship by treating structure and agency as mutually constitutive.

The framework also enables comparative analysis across creative sectors, platforms and national contexts. Future research might explore whether the five dimensions vary across various types of artists, including musicians, visual artists, writers, designers, dancers and performing artists and whether differences in platform architecture and governance produce different patterns of visibility and entrepreneurial opportunity. The relationship between sociocultural norms, institutional support, unequal access to digital resources, platform embeddedness and strategic engagement can be further explored through cross-cultural studies. The context-specificity and cross-platform variation of the relationships proposed by DSA could therefore be examined through comparative studies. DSA could also be examined in social-commerce environments where artistic production, social interaction and commercial exchange increasingly converge.

## 5 Proposed research questions and conceptual propositions

The five dimensions of DSA generate a set of research questions for future empirical investigation:

*RQ1:* How does platform embeddedness shape artrepreneurial practices and opportunities across digital platforms?

*RQ2:* How does algorithmically mediated visibility influence artistic recognition, legitimacy, audience access and artrepreneurial opportunity?

*RQ3:* How do artrepreneurs negotiate artistic authenticity, creativity and platform expectations when constructing digital identities?

*RQ4*: What is the role of networked social capital in artistic legitimacy, collaboration and opportunity for artrepreneurs?

*RQ5:* How artrepreneurs strategically respond to platform governance, algorithmic changes, audience feedback and emerging digital technologies?

These are the conceptual propositions that can be tested empirically to further clarify these research questions. These propositions are conceptual expectations that are designed to direct future empirical testing, but are not causal claims:

*P1:* Greater platform embeddedness is associated with greater dependence of artrepreneurial practices on platform-specific infrastructures, governance arrangements, and participation norms.

*P2:* Algorithmically mediated visibility influences the distribution of audience attention and may contribute to differences in cultural recognition, legitimacy, and artrepreneurial opportunity.

*P3:* Artistic identity is negotiated continually as artistic authenticity, the expectations of the audience, and platform-mediated norms of visibility and engagement are continually negotiated.

*P4:* Networked social capital strengthens the capacity of artrepreneurs to obtain relational resources, recognition, collaboration, and entrepreneurial opportunities within platform communities.

*P5:* Digital Strategic Engagement enables artrepreneurs to adapt to, negotiate, and potentially reshape the structural and algorithmic conditions of platform-mediated artistic entrepreneurship.

*P6:* The relationship between platform-mediated structures and artrepreneurial outcomes is jointly shaped by identity performance, networked social capital, and digital strategic engagement.

*P7:* Digital Strategic Engagement may amplify or alter the impact of algorithmic visibility, as artists react to the visibility mechanisms and audience response on platforms.

Lastly, the proposed perspective has implications beyond the academic realm. Cultural policymakers need to support artists in acquiring digital skills while maintaining and strengthening traditional artistic competencies. Similarly, digital literacy, platform awareness, community-building skills and ethical understanding of algorithmic systems should be integrated into arts entrepreneurship programmes that prepare artists for digitally mediated creative work. Platform companies also be encouraged to improve the transparency, accountability and fairness of their recommendation systems, which can significantly influence creative livelihoods.

These implications position Digital Sociology of Artrepreneurship not as a stand-alone entrepreneurship theory, but as a sociologically informed conceptual framework for understanding changes in the nature of creative work and entrepreneurial practice in digital society. Sociological research will become more and more vital in the future to account for how artists will navigate the new forms of digital labour, institutional control and entrepreneurial opportunity as the platform technologies continue to develop.

## 6 Conclusion

This Perspective has argued that artistic entrepreneurship in the platform society cannot be understood solely in terms of individual entrepreneurial activity or the use of social media as a medium for promotion. Platforms are becoming increasingly functioning as institutional spaces that define visibility, interaction, identity construction and access to entrepreneurial opportunities. DSA provides a framework for understanding this process through five interconnected dimensions: Platform Embeddedness, Algorithmic Visibility, Performative Artistic Identity, Networked Social Capital and Digital Strategic Engagement.

The contribution of DSA lies in connecting platform structures and algorithmic mechanisms with identity work, relational resources and strategic agency. Artrepreneurial outcomes are understood as emerging through the recursive interaction between structural conditions and situated action rather than as direct outcomes of individual agency or technology alone. The framework also highlights the interaction between platform-mediated visibility and existing inequalities, as well as the potential for digital technologies and artificial intelligence to transform creative practice and entrepreneurship.

The research questions and propositions outlined above can be examined across various creative sectors, platforms and cultural contexts, through future empirical studies. Hence, DSA is not proposed as a general theory of artistic entrepreneurship, but as a theoretically and sociologically informed conceptual approach for studying and analysing artistic entrepreneurial practices in digitally structured societies.

## Data Availability

The original contributions presented in the study are included in the article/supplementary material, further inquiries can be directed to the corresponding author.
